# Marketing of menthol cigarettes and consumer perceptions

**DOI:** 10.1186/1617-9625-9-S1-S2

**Published:** 2011-05-23

**Authors:** Joshua Rising, Lori Alexander

**Affiliations:** 1 Center for Tobacco Products, U.S. Food and Drug Administration, Rockville, MD, USA; 2 Editorial Rx, Inc, Orange Park, FL, USA

## Abstract

In order to more fully understand why individuals smoke menthol cigarettes, it is important to understand the perceptions held by youth and adults regarding menthol cigarettes. Perceptions are driven by many factors, and one factor that can be important is marketing. This review seeks to examine what role, if any, the marketing of menthol cigarettes plays in the formation of consumer perceptions of menthol cigarettes. The available literature suggests that menthol cigarettes may be perceived as safer choices than non-menthol cigarettes. Furthermore, there is significant overlap between menthol cigarette advertising campaigns and the perceptions of these products held by consumers. The marketing of menthol cigarettes has been higher in publications and venues whose target audiences are Blacks/African Americans. Finally, there appears to have been changes in cigarette menthol content over the past decade, which has been viewed by some researchers as an effort to attract different types of smokers.

## Review

The marketing of cigarettes is a significant expenditure for the tobacco industry; in 2006, the tobacco industry spent a total of $12.49 billion on advertising and promotion of cigarettes including menthol brands, which represent 20% of the market share [1].

This article explores the available literature on the perception of menthol cigarettes by consumers, the marketing of menthol cigarettes, and the association—if any—between the two. The following questions are explored here:

• How do youth and adult smokers perceive menthol cigarettes?

• Are there differences by racial or ethnic subgroup, or by gender?

° What is the history of the marketing of menthol cigarettes?

• What has been the impact of this marketing?

• What do publicly available internal tobacco company documents tell us about industry knowledge of consumer perceptions and any recent changes in menthol cigarette product design?

Summarized in this review are 35 articles found to have either direct relevance to these questions, or were used to provide relevant background information. Many of these articles were identified through a review of the literature conducted by the National Cancer Institute in 2009, published as “Bibliography of literature on menthol and tobacco” (http://cancercontrol.cancer.gov/tcrb/documents/menthol_bibliography_508.pdf). Search terms used were menthol cigarette(s); mentholated cigarette(s); menthol tobacco; mentholated tobacco; menthol smoker(s); menthol AND the following terms: addiction, nicotine, marketing, cancer, biomarkers, asthma, cardiovascular disease, heart disease, vascular disease, chronic obstructive lung disease, respiratory, environmental tobacco smoke, national health, health disparities, and minority health. Additional searches and sources, such as those identified through review articles, identified additional articles that were included as appropriate.

Of those articles that are in the NCI Bibliography but were not included, most were not directly relevant to this topic (e.g., they studied menthol as a chemical independent from tobacco smoke exposure, did not evaluate menthol as a separate variable). Some of those articles, however, were used to provide background information. Animal or in vitro research was included only to help explain human findings. Although a few review articles were used to make general statements and/or provide background information, most were not included in deference to original sources. Published abstracts were not included out of concern that, due to the lack of details, those studies could not adequately be assessed.

## Perceptions of menthol cigarettes

### Adult perceptions of menthol cigarettes

Only three studies on adult perceptions of menthol cigarettes were identified. Surveys and focus groups conducted by the tobacco industry (which are reviewed in the section on publicly available internal tobacco industry documents) also provided some information on consumer perceptions.

Hymowitz et al [2] surveyed 213 menthol smokers and asked why they smoked menthol cigarettes (Table 1)

**Table 1:** Reasons for smoking menthol cigarettes (1)

	White respondents (*n* = 39)	African American respondents (*n* = 174)
Menthol cigarettes taste better than regular non-menthol cigarettes	74%	83%
Menthol cigarettes are more soothing to my throat than regular non-menthol cigarettes	51%	52%
I can inhale menthol cigarettes more easily than regular non-menthol cigarettes	21%	48%
I can inhale menthol cigarettes more deeply than regular non-menthol cigarettes	10%	33%
I always smoked menthol cigarettes	39%	63%
Members of my family smoke menthol cigarettes	15%	30%
My friends that smoke, smoke menthol cigarettes	18%	41%
Menthol cigarettes suit my self-image better than regular non-menthol cigarettes	5%	14%
Menthol cigarettes are better for you than regular non-menthol cigarettes	5%	7%
Most of the advertising I see is for menthol cigarettes	3%	10%

The study did not assess the statistical significance of the differences between White and Black/African American smokers. More than half of all respondents, regardless of race, stated that their reasons for smoking menthol cigarettes included that they “taste better” and are “more soothing” as compared to non-menthol cigarettes. Of note, 7% of African American and 5% of White smokers stated that one reason for smoking menthol cigarettes was that they “are better for you,” and 10% of African American smokers cited the advertising of these products as a reason for smoking them.

Richter and colleagues explored health risk perceptions in two studies using focus groups. In the first, Black/African American men and women (ages 45–64 years) who smoked menthol cigarettes participated in small-group discussions [3]. Each discussion group included nine individuals who discussed a different topic; one such topic was the health effects of menthol. Individuals in this group described menthol cigarettes as “refreshing,” “soothing,” and “smooth” and non-menthol cigarettes as “strong” or “harsh.” Some of the group participants attributed greater health problems to non-menthol cigarettes. When participants were asked to rank 10 packs of menthol or non-menthol brands in order of least to most dangerous, they consistently placed the menthol brands in the intermediate position, between “light” and “slim” cigarettes (perceived as least dangerous) and full-flavor non-menthol cigarettes.

Also evaluated in this study were the smokers’ perceptions of advertising strategies. The majority of the participants agreed that menthol cigarettes were predominantly featured in Black publications, and that most cigarette advertising and marketing in their communities were for menthol brands, with minimal advertising of non-menthol brands. Some participants thought that tobacco companies targeted menthol cigarettes to Black/African American communities (and non-menthol cigarettes to White communities) and believed that advertising played a role in what brands were sold in an area. They acknowledged, however, that many communities are racially/ethnically mixed, and that taste is an important factor in selecting a type of cigarette. Participants also thought that the predominant themes of ads for menthol and non-menthol cigarettes differed. They said that ads for non-menthol cigarettes featured outdoor scenes, adventure, and athletic outdoor activities. In contrast, they used terms such as “relaxed,” “kicked back,” and “cool” to describe ads for menthol cigarettes and also noted that these ads depicted urban, hip-hop, or party scenes. One participant noted, “The message is smoke menthol and you’ll have fun.”

In another study by Richter and colleagues, 16 focus groups of White, Hispanic/Latino, or Black/African American young adult (aged 18–22 years) current smokers were asked to discuss their knowledge and health perceptions of cigarettes and nontraditional tobacco products, and to compare the safety of light, non-menthol, and menthol cigarettes to one another using terms of “safer than,” “the same risk as,” and “more harmful” [4].

The researchers found that “most of the non-Hispanic White participants rated light cigarettes as safer than menthol and menthol as more harmful than light. Comparisons between regular and menthol cigarette varieties were not consistent when the order of presentation was varied.” When examining the responses of Hispanic/Latino participants, the researchers found, “In comparisons with menthol and regular cigarettes, most of the non-in-college participants chose the more harmful ratings [for non-menthol cigarettes], regardless of the order of presentation of the products.” The researchers found with African Americans, “light cigarettes [were] rated as either safer or the same risk as menthol cigarettes. In comparisons with menthol and regular cigarettes, most college and not-in-college participants chose the same risk or more harmful ratings, regardless of the order of presentation of the products” [4]. Thus, neither menthol nor non-menthol cigarettes were consistently considered more harmful. This study did not control for type of cigarette smoked in the analysis.

A study by Wachowski and colleagues used the 2005 New Jersey Adult Tobacco Survey to investigate smokers’ risk perceptions. Unlike the previously discussed findings, they found that 30.2% of menthol smokers believed that menthol cigarettes were more risky than non-menthol cigarettes, as compared to 25.9% of all respondents (including smokers and non-smokers). [5]

### Youth perceptions of menthol cigarettes

No articles were found that contained information on youth perceptions of menthol cigarettes.

## Marketing history

In general, marketing of a product includes branding (name and packaging), advertising and promotion, product placement, and pricing. All of these tactics have been used to strategically market tobacco products, including menthol cigarettes. The brand names for some of the first menthol cigarettes were chosen to reflect “coolness,” the characteristic that was thought to set menthol cigarettes apart. Among the early brand names were Snowball, Skis, and Penguin [6]. The Penguin name was replaced by Kool, but the mascot, Willie the Penguin, remained. Advertisements expanded on the theme of coolness. Sutton and Robinson [7] evaluated advertisements for several brands of menthol cigarettes and identified four distinct types of messages: healthy/medicinal; fresh, refreshing, cool, and crisp; youthful, silliness, and fun; and ethnic awareness.

### Healthy/medicinal messages

Authors of reviews and commentaries (drawn from review of publicly available internal tobacco industry documents) have noted that menthol cigarettes were described in ads during the 1940s and early 1950s as “smooth,” “cool,” and “healthier” [6-8]. Ads for early brands of menthol cigarettes suggested that smokers should use this type of cigarette when their throats were irritated or they had a cold, reinforcing the concept of the menthol cigarette having a medicinal property [7]. In promoting the Kool brand of cigarettes, Willie the Penguin was often dressed as a doctor in print, television, and point-of-sale advertisements [6].

In a study of tobacco advertisements, Samji and Jackler [9] collected and reviewed several thousand ads that had appeared in print between 1920 and 1954, focusing on ads that depicted physicians and/or had a theme of throat health. Two of the cigarette brands featured in the collection of advertisements were Spuds (the first patented menthol cigarette) and Kool. Early advertisements for Spuds depicted the “smoke zone” as being the same as the “colds zone”; one such ad read: “Smoke soothing smoke, Spud Smoke, for the good and comfort of your nose and throat” [9]. The slogans used to advertise Spuds and Kool (and later menthol brands as well) included health messages that capitalized on the benefits of menthol as a home remedy for throat irritation, colds, and other respiratory illnesses (Table 2) [7,9].

**Table 2:** Health-Related Messages in Slogans in Early Advertisements of Two Brands of Menthol Cigarettes, 1920–1954: Spuds and Kool [9]

Cigarette Brand	Slogans
Spuds	“When your throat is irritated, change to Spuds.” “Nose or throat congested? It’s time to change to Spuds.” “Throat sore? Time to give it a rest.” “Smoke like a chimney? Who cares! Your mouth will taste clean as a whistle.”

Kool	“Doctors…agree that Kools are soothing to your throat.” “For your throat’s sake—switch from ‘hots’ to Kools.” “Your throat will like the change. The mild menthol is definitely refreshing.” “Those holiday throats need a carton of Kools.” “Keep a clear head with Kools. All the signs seem to point to a tough winter: cold, ice, chills and sniffles. Why not play it safe and smoke Kools?” “Has a stuffed-up head killed your taste for smoking? Light a Kool. The mild menthol gives a cooling, soothing sensation…leaves your nose and throat feeling clean and clear.” “There is just enough menthol in Kools to soothe your throat and refresh your mouth no matter how hot the weather gets—no matter how hard and how long you smoke.”

### Fresh, refreshing, cool, and crisp; youthful, silliness, and fun

According to literature reviewing internal tobacco industry documents that are now publicly available, the health properties of menthol were replaced in advertising with phrases such as “cool,” “clean,” “crisp,” and “fresh” beginning in the late 1950s and continuing through the 1960s. Images in print advertisements reinforced these words, depicting rain forests, waterfalls, woods, and streams. Beginning in the 1970s and 1980s, other advertising campaigns focused on youthfulness, silliness, and fun [7]. The Newport campaign, “Alive with Pleasure” is one example of this latter theme. Sutton and Robinson go on to state that “the central theme of Newport advertising is obvious: ‘Kids just want to have fun.’…The advertising messages over the past 30 years have stressed that smoking a Newport is part of youth, part of having pleasure in life, part of having a good time” [7].

A summary of a focus group conducted on behalf of the tobacco industry noted that Newport was considered to be a cigarette for young people who get “high on life” [7]. Images were primarily of people having fun and taking part in youthful activities. When Balbach et al [10] reviewed advertisements of menthol cigarettes to the Black/African American population, they identified escape/fantasy and nightlife fun as two of the three primary images featured in the ads.

### Ethnic awareness campaigns

Several tobacco researchers have provided an overview of the marketing of menthol cigarettes to the Black/African American population, noting that the marketing strategy followed a course similar to the social evolution of the Black/African American community [6,7,10,11]. This marketing approach began after World War II, when Black/African American individuals began moving into urban areas and the marketing became more aggressive to reach low-income Black/African American individuals in inner city communities, with menthol cigarettes promoted as “sophisticated” and “cool” [6,7,10,11]. The “cool” psychologic identity of being a smoker of menthol cigarettes is one of several factors that influence the choice of this type of cigarette, according to Castro [12]. Several tobacco companies engaged ethnic marketing firms to help them develop specific marketing strategies to reach the Black/African American population [11].

Later advertisements featured Black/African American models and spokespersons (especially leading athletes and entertainers), Afro hairstyles; popular music (soul, jazz, hip-hop) and other content drawn from Black/African American pop culture [6,7,13].

In their study of ads appearing in Black/African American publications during 1989–1990, Balbach et al [10] also found that ads featured images conveying a sense of fun and of being suave and sophisticated, and they identified predominant themes of an escape/fantasy setting, images of expensive objects, and a nightlife setting (100%, 73.7%, and 57.7%, respectively); the escape/fantasy and nightlife settings continued in 1999–2000 (71.9% and 77.8%, respectively), although there was a substantial decrease in the use of images of expensive objects (15.9%).

## Data on marketing

### Marketing to Black/African American individuals

Research that has been done on marketing to specific racial or ethnic subgroups has compared marketing to a specific subgroup with marketing to White individuals. In order to explore this area, published studies have looked at the following types of marketing: magazines, billboards, point-of-sale, and promotions. Little research has been done on the impact of this marketing.

Cummings et al [14] reviewed full-page cigarette ads appearing from June 1984 to May 1985 in seven different magazines, with four directed primarily at a White audience: *Newsweek*, *Time*, *People*, and *Mademoiselle*, and three directed primarily at a Black/African American audience: *Jet*, *Ebony*, and *Essence*. The researchers found that the ads for cigarettes in the three publications for Black/African American audiences were focused on menthol cigarettes (83.4% in *Jet*, 59.1% in *Ebony*, and 65.7% in *Essence*). In contrast, the cigarette ads in magazines directed at White audiences were much less likely to be for menthol cigarettes (24.6% in *People*, 5.1% in *Time*, and 4.9% in *Newsweek*).

Data from a later study found a similar pattern, with more ads for menthol cigarettes in publications designed for Black/African American individuals. In their review of 274 cigarette ads published between January 1998 and August 2002 in 54 issues of *People* and 56 issues of *Ebony*, Landrine et al [8] found a significant difference in the prevalence of ads for menthol cigarettes, with 67.2% of the cigarette ads in *Ebony* being for menthol cigarettes, compared with 17.3% in *People* (p < .0005). Stepwise logistic regression analysis indicated that *Ebony* was 9.8 times as likely as *People* to contain an ad for menthol cigarettes.

Balbach et al [10] also compared cigarette ads in popular magazines with primarily Black/African American or White readers. These researchers compared 379 ads for cigarettes (RJ Reynolds brand) published in *Jet*, *Ebony*, and *Essence* with those published in *People Weekly* at two time points spanning a decade (in 1989–1900 and again in 1999–2000). During the two time periods, virtually all of the RJ Reynolds ads in *Jet*, *Ebony*, and *Essence* were for menthol cigarettes (100% and 97.7%), compared with 31.6% and 0% in *People Weekly* (p < .001).

Altman et al [15] conducted an analysis of 901 billboards in one urban setting and found that tobacco was the leading product advertised (19% of all billboards), with menthol cigarettes advertised in 13% of all tobacco billboards. The proportion of tobacco billboard ads was significantly higher in Black/African American neighborhoods than in White neighborhoods (24% vs. 17%; p < .03), and Black/African American neighborhoods were also more likely than White neighborhoods to contain billboards with ads for menthol cigarettes (22% vs. 11%; p < .01).

The findings of a study of point-of-sale advertising study in one community demonstrated the marketing of menthol cigarettes to the Black/African American population. Laws et al [16] found that the percentage of ads for menthol cigarettes was highest (32.3%) in stores selling tobacco products in a predominantly Black/African American urban community, compared with 10% of ads in non-minority neighborhoods. Both the Laws [16] and the Altman [15] study are limited in that they only examined advertising in one geographic area.

Promotional offers on cigarettes (e.g., coupons, two-for-one offers, retailer discounts), which represented three-quarters of the tobacco industry’s marketing expenses in 2002, are another tactic that can focus on specific populations [13]. White et al [13] analyzed data from 4,618 current smokers who responded to the population-based 2002 California Tobacco Survey (a random-digit-dialed survey). Among all respondents, smokers of menthol brands used promotional offers more often than smokers of the two other leading (non-menthol) brands (57.1% [menthol brand] vs. 49.1% [Camel] and 34.8% [Marlboro]); this analysis did not control for age, race, or socioeconomic status. The researchers also found that among Black/African American individuals, those who smoked menthol cigarettes were more likely to use promotional offers than those who smoked non-menthol cigarettes (65.4% vs. 28.7%).

Finally, one cross-sectional survey examined the influence of tobacco advertising; the survey was conducted among adult Black/African American smokers in a low socioeconomic, urban area of Los Angeles [17]. Approximately 69% of the 432 survey participants (115 men and 181 women) smoked menthol cigarettes. Participants were asked two questions to evaluate their exposure to advertising:

• When you were a child, the ads you saw or heard most often were for menthols or non-menthols?

• Currently, the ads you see most often are for menthols or non-menthols?

Women who were exposed to ads for menthol cigarettes in their childhood had a higher odds ratio (1.72) for currently smoking menthol cigarettes than women who were not exposed, though this was not statistically significant. Men who had been exposed to ads for menthol cigarettes in their childhood had a lower odds ratio (0.61) for currently smoking menthol cigarettes than men who were not exposed, though this was also not statistically significant. Finally, the odds ratios were higher for both men and women to be more likely to smoke menthol cigarettes if they were currently exposed to advertising of menthol cigarettes, but these results were not statistically significant either.

### Marketing to Hispanic/Latino individuals

Research on marketing to Hispanic/Latino populations has been examined in three studies looking at the advertising of menthol cigarettes to Hispanic/Latino populations. All of these studies were also discussed in the last section, as they also examined the advertising of menthol cigarettes to Black/African American populations.

The study by Landrine et al [8] included 31 issues of the Spanish version of *People* in its comparison of cigarette ads. The Hispanic/Latino audience did not appear to be a large focus of cigarette ads overall, with a mean of 1.58 ads per issue (compared with 1.87 ads per issue of *People* and 2.25 ads per issue of *Ebony*). Although most ads were for non-menthol cigarettes, the Spanish version of *People* was 2.6 times more likely than the English version of People to contain ads for menthol cigarettes [8]. The authors concluded that the tobacco industry appeared to be using similar strategies to market to the Hispanic/Latino population as had been used with the Black/African American population.

In the study of billboards by Altman et al [15], Hispanic/Latino neighborhoods had significantly more tobacco ads as compared to White neighborhoods or Asian neighborhoods (25% vs. 17% and 14% respectively; p < .03). Hispanic/Latino neighborhoods were also significantly more likely to contain billboards with ads for menthol cigarettes as compared to White or Asian neighborhoods (17% vs. 11% and 10%, respectively; p < .01).

Further information is provided by the study of point-of-sale advertising by Laws et al [16]. The rates of advertising for menthol brand cigarettes were higher in two predominantly Hispanic/Latino communities (18.1% and 14.8%) than in nonminority neighborhoods (10%).

### Marketing to women

Little published research was found on the advertising of menthol cigarettes to women. The bibliography includes two reviews about tobacco use among women, but neither had menthol-specific advertising information.

One article based on review of internal tobacco industry documents noted that ads for menthol cigarettes were often designed to appeal to women, with images of romantic couples, flowers, and springtime [7].

### Marketing to youth and young adults

Youth are an important population for the tobacco industry, as smoking initiation most often occurs before the age of 21. Only three articles describing research on the marketing of menthol cigarettes to youth and young adults were identified [18-20].

Before describing this research on menthol cigarette marketing to youth, it is important to note the substantial role of tobacco advertising as an influencing factor for youth tobacco users. According to several cross-sectional and longitudinal surveys, adolescents (aged 12–17 years) have high receptivity to tobacco advertisements, and, in turn, high receptivity is associated with enhanced appeal of smoking, smoking initiation, and smoking progression (an increase in smoking behavior) among youths [21-25]. Specifically, surveys have shown that adolescents who report a high level of exposure to cigarette advertising are up to twice as likely to be cigarette smokers [22], and that receptivity to advertising is a stronger influencing factor in determining susceptibility to initiating smoking than exposure to peer or family smokers or sociodemographic variables [23]. Receptivity to advertising has varied according to race/ethnicity, with the highest receptivity among non-Hispanic White adolescents, and lower levels among Hispanic/Latino, Black/African American, and Asian American/Pacific Islander youth [26,27] In addition, multiple studies have shown that young smokers (aged 13–18 years) use the most extensively advertised cigarette brands [28-30].

The strongest evidence of advertising as an important influence on youth tobacco use is a systematic review of longitudinal studies that found exposure to tobacco advertising and promotion to be associated with increased likelihood of smoking initiation among adolescents [31]. However, advertisements for menthol cigarettes were not specifically evaluated in these surveys.

One study in the bibliography included a component to evaluate youth’s perceptions of menthol cigarettes. In this 2008 study, five focus groups were conducted among Black/African American children (mean age, 12–13 years) in Washington, D.C.; the total sample size was small (28 children), with three to eight children in each of 5 focus groups [19]. Only four of the 28 children said that they had ever smoked. Limited findings were presented in the report, and menthol cigarettes were not specifically discussed. As part of some group discussions, the authors asked participants to recall specific tobacco marketing campaigns, and the following is an image recalled by one participant of a menthol cigarette brand: “They in the, the Kool magazine, they always have black people smoking…they were smoking and having fun…just standing up, like laughing” [19].

One study of advertising directed at youth was a cross-sectional study of 3,151 advertisements of tobacco products in 184 retail stores in Hawaii [18]. Advertisements were weighted by size (small, medium, large) based on the hypothesis that larger ads may have greater visual impact. Among the four brands that accounted for two-thirds of the advertisements, the cigarette with the largest number of total ads based on weighted data (848 ads) was a menthol brand (Kool). Kool also had the most outdoor ads, and, overall, approximately 31% of stores within 1,000 feet of a school or a playground had outdoor tobacco ads. Kool is the leading brand smoked by youth in Hawaii, suggesting an association between use of menthol cigarettes and advertising, but the study was not designed to determine the presence of a causal relationship.

The other study specifically looking at the marketing of menthol cigarettes to younger individuals examined the perceived age of models in ads for menthol and non-menthol cigarettes [20] In that study, the researchers selected 50 ads (from 65 publications) that were judged to include a model whose face was “clearly visible.” Of the 12 brands of cigarettes in the ads, three were menthol (Newport, Newport Lights, and Kool Milds). A total of 22% of the models were perceived to be aged 18–24 years. Among the brands with the highest percentage of models perceived to be younger than 25 years were Kool Milds (5 of 10 models) and Newport Lights (1 of 2 models). On average, ads for menthol cigarettes tended to have models who were perceived as looking younger (mean: 25.7 years) than models in ads for non-menthol cigarettes (mean: 31.9 years). Ads for menthol cigarettes also tended to appear in magazines that had more youthful audiences. The average audience age was 31.1 for publications with Newport Lights ads and 31.3 for publications with Newport and Kool Milds ads, while the audience age for non-menthol cigarette ads ranged from 31.3 to 41.0, with the exception of Lucky Strike Lights (average audience age = 28.5).

## Research on publicly available internal tobacco industry documents

Four published articles were identified that examined publicly available internal tobacco industry documents for information on consumer perceptions of menthol cigarettes and on the impact of tobacco industry marketing of menthol cigarettes [19,32-34].

### Adult perceptions of menthol cigarettes

In reviewing menthol cigarette use, Giovino et al [32] used a quote from a focus group study done for Philip Morris in 1968:

“There are indications that menthols tend to be considered generally ‘better for one’s health.’ That impression refers not only to the health of the respiratory tract, but the whole organism. The majority view is that menthols are ‘less strong’ than regular cigarettes, and that a cigarette which is ‘less strong’ is better for a person’s health.”

An RJ Reynolds document from 1977 discussed racial differences in the perception of menthol cigarettes; it highlighted that Black/African American menthol cigarette smokers were more likely than White menthol cigarette smokers to believe that menthol cigarettes were “better if you smoke a lot,” “lower in tar and nicotine,” “less likely to make you cough,” “better when you have a cold,” and “less irritating to the throat” [32].

An internal tobacco industry document from 1986 also described differing preferences among menthol smokers, and that not all menthol cigarette smokers desired the same level of menthol in their cigarettes [33].

“All three major brands (Salem, Kool, Newport) built their franchise with YAS [younger adult smokers]…using a low menthol product strategy. However, as smokers acclimate to menthol, their demand for menthol increases over time…Responsive brands whose strategy is to maximize franchise value invariably increase menthol levels over time.”

Another quotation from a 1987 document made a similar point:

“A product having a moderate to high menthol taste will usually be rejected by starters, while the same level will be quite acceptable to established menthol smokers” [34].

### Youth and young adult perceptions of menthol cigarettes

Kreslake et al examined publicly available tobacco company documents for information on the marketing of menthol cigarettes to youth and young adults. In an RJ Reynolds document from 1987, they highlighted a quotation that showed knowledge that younger smokers preferred lower levels of menthol in cigarettes: “The want for less menthol does indeed skew younger adult.” Furthermore, Kreslake et al cite documents that they clain demonstrate that RJ Reynolds considered the low menthol levels one of the main reasons that Newport cigarettes were so popular among younger smokers [33].

In their review of publicly available internal tobacco industry documents, Johnson et al [19] found that tobacco companies conducted market research to better understand the smoking patterns of young Black/African American individuals and sought strategies to enhance the prevalence of smoking, and especially the smoking of menthol cigarettes, in this population.

### Recent changes in menthol levels of cigarettes

Based on a review of publicly available internal tobacco company documents, Kreslake et al outlined a number of changes made to the menthol levels in menthol cigarettes between 2000 and 2007 [33]. They documented that two new brands of menthol cigarettes were introduced with low levels of menthol, that two existing brands lowered their menthol levels between 2000 and 2007, and that one brand increased its menthol level. They concluded their review by stating, “We found evidence that the tobacco industry… introduced new menthol brands to gain market share, particularly among adolescents and young adults.”

## Conclusions

The marketing and advertising of menthol cigarettes is a possible contributing factor to the higher rates of menthol cigarette use among several population subgroups. However, it is difficult to draw definitive conclusions because of the limited research that is available and the cross-sectional nature of the research (which can demonstrate associations but are limited with regard to assessing causality). Furthermore, limitations of the studies that have been published include retrospective designs, small sample sizes, a small geographic survey area, and reliance on focus groups.  This makes it difficult to generalize the research findings.

The research outlined above does support the following conclusions:

• Research studies and reviews of publicly available internal tobacco industry documents suggest that menthol cigarettes may be perceived to be safer choices than non-menthol cigarettes.

• There is significant overlap between the themes of menthol cigarette campaigns and consumer perceptions of menthol cigarettes.

• Marketing of menthol cigarettes is higher in publications/venues whose target audiences are Black/African Americans.

• Publicly available internal tobacco industry documents differentiate the preferences of younger smokers with those of experienced smokers, with younger smokers preferring lower levels of menthol than experienced smokers.

• There have been changes in cigarette menthol content over the past decade as some brands have moved towards lower levels of menthol and others toward higher levels of menthol. This has been viewed as the tobacco industry modifying the menthol cigarette in order to attract different types of smokers, such as inexperienced versus experienced smokers.

## Competing interests

The authors declare that they have no competing interests.
